# Oncologic Long-Term Results of Robot-Assisted Minimally Invasive Thoraco-Laparoscopic Esophagectomy with Two-Field Lymphadenectomy for Esophageal Cancer

**DOI:** 10.1245/s10434-015-4544-x

**Published:** 2015-05-29

**Authors:** P. C. van der Sluis, J. P. Ruurda, R. J. J. Verhage, S. van der Horst, L. Haverkamp, P. D. Siersema, I. H. M. Borel Rinkes, F. J. W. ten Kate, R. van Hillegersberg

**Affiliations:** Department of Surgery, University Medical Center Utrecht, Utrecht, The Netherlands; Department of Gastroenterology and Hepatology, University Medical Center Utrecht, Utrecht, The Netherlands; Department of Pathology, University Medical Center Utrecht, Utrecht, The Netherlands; Department of Surgery, G04.228, University Medical Center Utrecht, Utrecht, The Netherlands

## Abstract

**Background:**

Open transthoracic esophagectomy is the worldwide gold standard in the treatment of resectable esophageal cancer. Robot-assisted minimally invasive thoraco-laparoscopic esophagectomy (RAMIE) for esophageal cancer may be associated with reduced blood loss, shorter intensive care unit (ICU) stay, and less cardiopulmonary morbidity; however, long-term oncologic results have not been reported to date.

**Methods:**

Between June 2007 and September 2011, a total of 108 patients with potentially resectable esophageal cancer underwent RAMIE at the University Medical Centre Utrecht, with curative intent. All data were recorded prospectively.

**Results:**

Median duration of the surgical procedure was 381 min (range 264–636). Pulmonary complications were most common and were observed in 36 patients (33 %). Median ICU stay was 1 day, and median overall postoperative hospital stay was 16 days. In-hospital mortality was 5 %. The majority of patients (78 %) presented with T3 and T4 disease, and 68 % of patients had nodal-positive disease (cN1–3). In 65 % of patients, neoadjuvant treatment (chemotherapy 57 %, chemoradiotherapy 7 %, radiotherapy 1 %) was administered, and in 103 (95 %) patients, a radical resection (R0) was achieved. The median number of lymph nodes was 26, median follow-up was 58 months, 5-year overall survival was 42 %, median disease-free survival was 21 months, and median overall survival was 29 months. Tumor recurrence occurred in 51 patients and was locoregional only in 6 (6 %) patients, systemic only in 31 (30 %) patients, and combined in 14 (14 %) patients.

**Conclusion:**

RAMIE was shown to be oncologically effective, with a high percentage of R0 radical resections and adequate lymphadenectomy. RAMIE provided good local control with a low percentage of local recurrence at long-term follow up.

**Electronic supplementary material:**

The online version of this article (doi:10.1245/s10434-015-4544-x) contains supplementary material, which is available to authorized users.

In 2008, an estimated 482,300 people were diagnosed with esophageal cancer, and 406,800 patients died of the disease worldwide.^1^ Radical esophagolymphadenectomy is the cornerstone of the multimodality treatment with curative intent.^2^–^5^

Worldwide, open transthoracic esophagectomy is the preferred surgical approach for esophageal cancer, allowing en bloc resection of the tumor with the surrounding paratracheal, subcarinal and paraesophageal lymph nodes.^6^,^7^ However, the percentage of cardiopulmonary complications associated with the open transthoracic approach is high (50–70 %).^6^

Minimally invasive esophagectomy (MIE) was designed to reduce surgical trauma, resulting in lower rates of morbidity and mortality. With regard to MIE, a review of the literature shows a substantial decrease in blood loss, postoperative complications, and days of hospital stay, with comparable short-term oncologic results.^8^–^13^ These results were confirmed in a recently published randomized controlled trial where MIE was compared with open transthoracic esophagectomy.^14^ However, open transthoracic esophagectomy remains the gold standard worldwide for the treatment of esophageal cancer.^7^

In 2003, robot-assisted minimally invasive thoraco-laparoscopic esophagectomy (RAMIE) was developed at the University Medical Center Utrecht (UMC Utrecht), Utrecht, The Netherlands.^15^ Robot-assisted thoraco-laparoscopic esophagectomy facilitates complex minimally invasive procedures with an enlarged, three-dimensional (3D) field of view. The articulated instruments facilitate dissection with seven degrees of freedom.^13^,^15^–^18^

From our first experience, reported in 2006 and 2009, it was concluded that RAMIE is a feasible and safe technique, associated with reduced blood loss, shorter intensive care unit (ICU) stay, and a lower percentage of cardiopulmonary complications compared with literature reports of open transthoracic esophagectomy.^6^,^15^,^16^

Following these initial reports of RAMIE, the current article presents our subsequent series with a focus on long-term oncologic results.

## Methods

### Patients

Between June 2007 and September 2011, consecutive patients with potentially curative resectable esophageal cancer were operated on in the UMC Utrecht. In our institute, transthoracic esophagectomy is the standard treatment for patients with esophageal cancer. The standard neoadjuvant treatment for patients with esophageal adenocarcinoma was preoperative chemotherapy [epirubicin, cisplatin and capecitabine (ECC)].^19^ Patients with esophageal squamous cell carcinoma underwent preoperative chemoradiotherapy (carboplatin and taxol + 41.4 Gy).^20^ Data on surgical procedures were registered prospectively in the operating room. All complications and follow-up were registered in a prospective surgical database.

We prospectively recorded baseline characteristics and routine diagnostic work-up, including use and results of upper endoscopy, endoscopic ultrasound, computed tomography (CT) of the thorax and abdomen, and ultrasound of the neck region. Positron emission tomography (PET) scanning with fine-needle aspiration of the suspected lymph nodes was used at indication and recorded prospectively. All patients were discussed at a multidisciplinary oncology board meeting.

Patients received standard postoperative follow-up at the outpatient department. Patients visited the outpatient department at 6 weeks and 3, 6, 9, and 12 months in the first year, and in the second, third, fourth, and fifth year postoperatively. Patients received follow-up every 6 months. In case symptoms of tumor recurrence occurred, patients underwent a CT of the thorax and abdomen. All patients had at least 29 months of follow-up and were followed for 5 years postoperatively.

### Operative Procedure

The operative technique of thoraco-laparoscopic esophagectomy with two-field lymphadenectomy has been previously described.^15^,^16^ For the thoracic phase, the patient is positioned in the left lateral decubitus position, and tilted 45° towards the prone position. The trocar arrangement during the robot-assisted thoracoscopic and laparoscopic phases is shown in electronic supplementary Fig. S1.^15^ Robot-assisted esophagectomy included a thoracic lymphadenectomy, which included the right-sided paratracheal (lymph node station 2R), tracheobronchial (station 4), aortopulmonary window (lymph nodes in the window dorsal to the aortic arch, cranially to the left main bronchus up until the pulmonary artery, station 5), carinal (station 7), and perioesophageal (station 8) lymph nodes.^15^

The patient was placed in the supine position thereafter to facilitate a laparoscopic gastric mobilization, truncal lymph node dissection, and gastric tube formation with cervical hand-sewn end-to-side esophagogastrostomy.^21^

### Postoperative Management

Mechanical ventilation was continued until patients were transferred to the ICU, where they were extubated 2–3 h after ending the operation. After day 1, patients were transferred to the medium care unit (MCU) and then to the surgical ward on postoperative day 2.

All patients were placed on a nil-by-mouth routine with enteral tube feeding by a needle-catheter jejunostomy on the first 7 days postoperatively. Nasogastric tubes were routinely placed. No postoperative swallow tests were performed as the sensitivity rate of detecting leakage was considered to be too low to change postoperative decision making.^22^ In the absence of signs of anastomotic dehiscence, patients started with sips of water and the oral intake was gradually increased to solid food. There was no enhanced recovery program.

### Postoperative Complications

All complications were graded using the modified Clavien–Dindo classification (MCDC) of surgical complications. All reported complications were grade 2 and higher.^23^

### Pathological Analysis

The resected specimen was evaluated using a standard protocol, with emphasis on resection margins, tumor type, extension of the tumor, and the presence of lymph nodes. The 7th edition of the Union for International Cancer Control (UICC) was used for TNM classification, tumor grade, and stage grouping.^24^ The (circumferential) resection margins were evaluated using the College of American Pathologists (CAP) criteria.^25^

### Statistical Analysis

Statistical analysis was performed using SPSS version 20.0 (IBM Corporation, Armonk, NY, USA). A *p* value of <0.05 was considered to be statistically significant. All skewed continuous data were presented as medians and ranges. Survival time was calculated as the duration from the day of surgery to the date of death or date of last follow-up. Disease-free interval was calculated from the day of surgery to the day of definitive diagnosis of recurrent tumor.

## Results

Between June 2007 and September 2011, a total of 123 consecutive patients with potentially curative resectable esophageal cancer were eligible for transthoracic esophagectomy. In seven patients with locally advanced T4 tumors, an indication for open transthoracic esophagectomy was made preoperatively. Intraoperatively, irresectable disease was observed in 8 patients, leaving 108 patients eligible for RAMIE.

The baseline characteristics of patients are summarized in electronic supplementary Table S1. The patients included 76 men and 32 women, with a median age of 62 years (range 42–78) and a body mass index (BMI) of 26 (range 16–36 kg/m^2^ ). The majority of patients (78 %) were clinically staged as cT3 and higher, and 68 % of patients had clinically positive nodal disease (cN1–N3). Co-morbidity, consisting of a history of vascular, cardiac, pulmonary, and oncologic disease, was observed frequently within this cohort.

In 20 patients (19 %), conversion to an open transthoracic or open transhiatal procedure was needed. Conversion to thoracotomy (*n* = 11) was necessary due to bulky adhesive tumor in the mediastinum (*n* = 4), insufficient collapse of the right lung (*n* = 2), or inadequate thoracoscopic trocar position (*n* = 1). Four patients had bleeding that could not be controlled thoracoscopically (*n* = 4). One patient had bleeding from the bronchial artery, two patients had bleeding from the azygos vein, and one patient had an iatrogenic lung bleed. Conversion to a transhiatal procedure (*n* = 9) was necessary due to insufficient collapse of the right lung (*n* = 6), inadequate thoracoscopic port position (*n* = 1), pleural adhesions (*n* = 1), or enlarged right cardiac atrium (unusual anatomy) (*n* = 1).

Conversion of the laparoscopic abdominal phase to laparotomy was required in three patients due to bleeding that could not be controlled laparoscopically (*n* = 1), locally advanced tumor requiring total gastrectomy with colonic interposition (*n* = 1), or very low position of the greater curvature (*n* = 1). Patients who underwent intraoperative conversion did not statistically differ in baseline characteristics from patients who underwent a full RAMIE. There was a significant decrease in the percentage of conversions between the first group of 54 patients and the second group of 54 patients (13 [24 %] vs. 7 [13 %], respectively; *p* < 0.001).

### Operative Results

The operative data of 108 patients are shown in Table 1. The median duration of the total procedure was 381 min (range 264–550), and the thoracoscopic phase (88 patients) had a median duration of 175 min (range 108–241). There was a significant decrease in thoracoscopic operative time between the first group of 44 patients and the second group of 44 patients who completed the thoracic phase thoracoscopically (199 min vs. 166 min, respectively; *p* < 0.001).

**Table 1:** Patient operative data (*n* = 108)

Total operating room time [min; median (range)]	381 (264–636)
Thoracoscopic phase [median (range)]	175 (108–281)
Total blood loss [ml; median (range)]	340 (50–3800)
Conversion thoracoscopy	20 (19)
Reason for conversion	
Respiratory problems	8 (7)
Bleeding	4 (4)
Bulky tumor	4 (4)
Trocar problems	2 (2)
Pleural adhesions	1 (1)
Unusual anatomy	1 (1)
Conversion laparoscopy	3 (3)
Reason for conversion	
Advanced tumor	1 (1)
Bleeding	1 (1)
Unusual anatomy	1 (1)

Data are expressed as *n* (%) unless otherwise specified

### Postoperative Results

Postoperative data are summarized in Table 2. An uncomplicated postoperative course was observed in 37 (34 %) patients, and pulmonary complications were most common. Pneumonia was diagnosed and treated in 36 (33 %) patients, and anastomotic leakage of the esophagogastrostomy was seen in 20 (19 %) patients, of whom 6 (6 %) also had intrathoracic manifestation. Chylothorax was seen in 19 (18 %) patients; in 15 of these patients the leakages were low-volume and could be treated conservatively, showing that the leakage was only from small side branches of the thoracic duct.

**Table 2:** Postoperative data (*n* = 108)

Uncomplicated procedures	37 (34)
Complications	71 (66)
Pulmonary	36 (33)
Pneumonia	36 (33)
Atelectasis	6 (6)
Anastomotic leakage	20 (19)
Intrathoracic manifestations	6 (6)
Chylothorax	19 (18)
Vocal cord paralysis^a^	10 (9)
Cardiac	10 (9)
Atrial fibrillation	9 (8)
Myocardial infarction	1 (1)
Wound infection	7 (6)
Thromboembolic event	6 (6)
Pneumothorax	6 (6)
Other^b^	3 (3)
In-hospital death	5 (5)
ICU stay [days; median (range)]	1 (1–76)
Hospital stay [days; median (range)]	16 (9–123)

Data are expressed as *n* (%) unless otherwise specified

*ICU* intensive care unit

^a^ 8 temporary, 2 permanent

^b^ 1 omentum necrosis, 1 tracheoesophageal fistula, 1 bleeding

Vocal-cord paralysis occurred in ten (9 %) patients, and paralysis was temporary in eight of these ten patients. The permanent recurrence paralysis rate was 2 %. Wound infections were seen in seven (6 %) patients; five patients were diagnosed with a cervical wound infection, of whom one patient also had a thoracic wound infection. The remaining two patients had abdominal wound infections. Postoperative pneumothorax requiring additional chest tube placement was seen in six (6 %) patients, and thromboembolic complications were seen in 6 % of patients.

Patients were ventilated at the ICU for a median of 0 days (range 0–64). Median ICU stay was 1 day (range 1–76) and overall postoperative hospital stay was 16 days (range 9–123). In-hospital mortality was 5 % (four patients). One patient died from a myocardial infarction, one from a tracheo–neo-esophageal fistula, one from anastomotic leakage with respiratory insufficiency, and one from a mediastinal septic bleed following anastomotic leakage.

### Histopathological Results

An overview of the histopathological results is shown in Table 3. The majority of tumors were adenocarcinomas (78 %). In ten (9 %) patients, no viable tumor cells were detected in the resected specimen, corresponding to a pathological complete response (pCR) rate to neoadjuvant therapy of 14 %. The majority of tumors were located in the distal esophagus or at the gastroesophageal junction (GEJ) (85 %). In 102 (94 %) patients a radical resection (R0) was achieved. No gross irradical resections (R2 resections) were performed. In 108 operations, 2794 lymph nodes were retrieved, and the median number of lymph nodes was 26 (range 5–53). In total, 264 positive lymph nodes were dissected, with a median of one positive lymph node (range 0–22). The distribution of dissected lymph nodes is shown in electronic supplementary Fig. S2. In total, 15 % of all patients had lymph node metastases located at the subcarinal level and higher.

**Table 3:** Histopathological data (*n* = 108)

Histological type	
Adenocarcinoma	78 (72)
Squamous cell carcinoma	20 (19)
No viable tumor cells	10 (9)
Site of tumor	
Mid or upper esophageal	16 (15)
Lower esophageal and GEJ	92 (85)
Radicality	
R0	103 (95)
R1	5 (5)
No. of retrieved LNs [median (range)]	2794 [26 (5–57)]
No. of positive LNs [median (range)]	264 [1 (0–22)]
Pathological T stage (%)	
pT0	10 (9)
pT1	20 (19)
pT2	11 (10)
pT3	65 (60)
pT4a	2 (2)
Pathological N stage (%)	
pN0	48 (44)
pN1	30 (28)
pN2	20 (19)
pN3	10 (9)

Data are expressed as *n* (%) unless otherwise specified

*GEJ* gastroesophageal junction, *LNs* lymph nodes

### Recurrence and Outcome

**Fig. 1:**
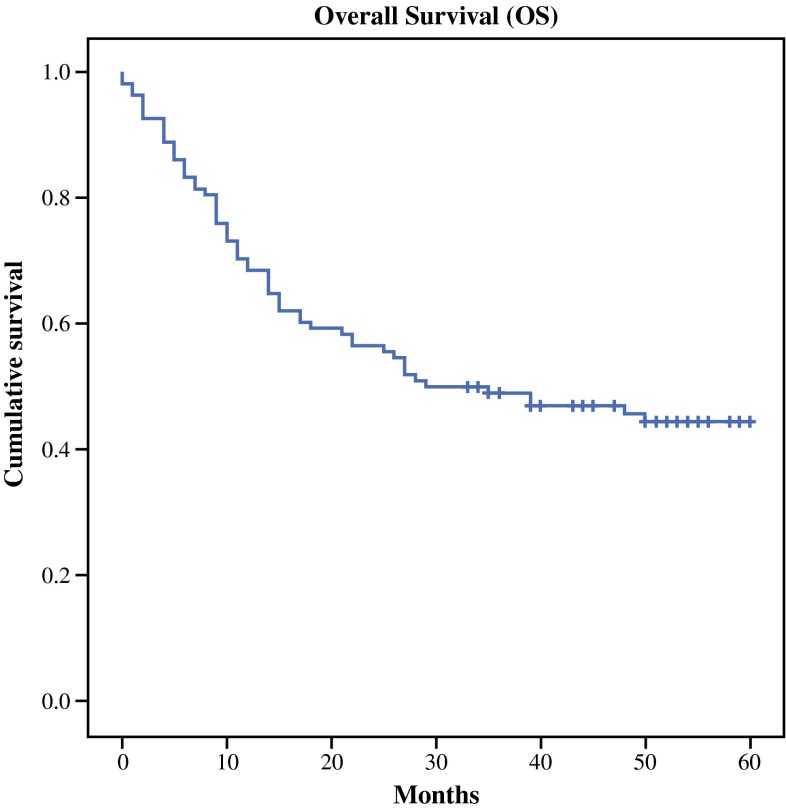
Kaplan–Meier curves for overall survival (months)

At the time of analysis, a median of 58 months after surgery, all patients had undergone esophagectomy at least 29 months previously. No patients were lost to follow-up, and median overall survival was 29 months. Kaplan–Meier curves for overall survival are shown in Fig. 1. Overall 5-year survival was 42 %.

**Fig. 2:**
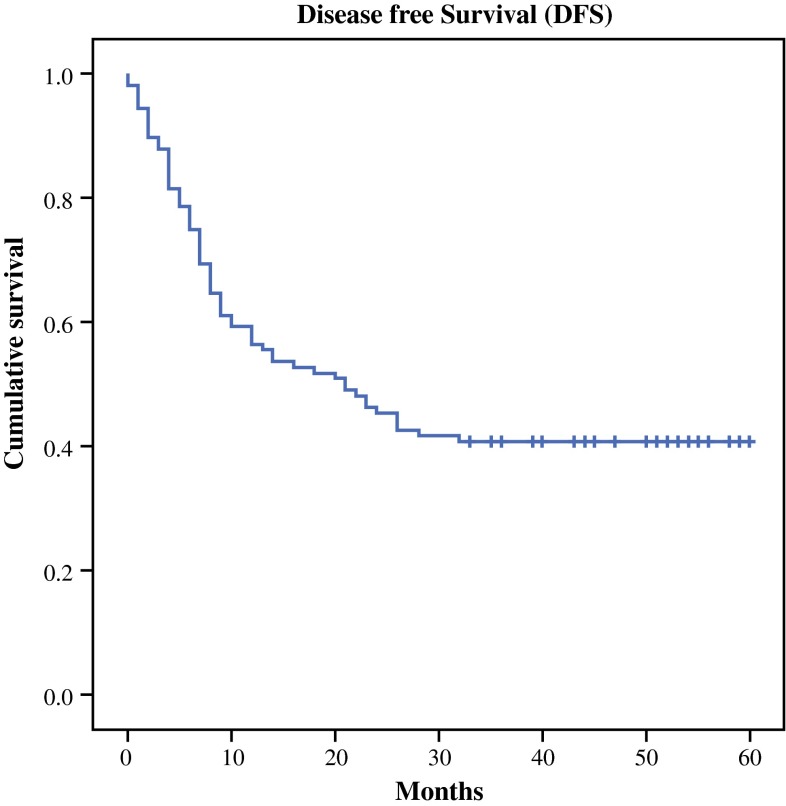
Kaplan–Meier curves for disease-free survival (months)

Of 108 patients, 5 died postoperatively; therefore, 103 patients were included in the recurrence analysis. Median disease-free survival was 21 months. In 42 patients (52 %), no signs of recurrent disease were observed after a median follow-up of 34 months. The remaining 39 patients developed symptomatic recurrent disease. In 52 of 103 patients (51 %), no signs of recurrent disease were observed after a median follow-up of 34 months. The remaining 51 patients developed symptomatic recurrent disease. The first site of symptomatic tumor recurrence was locoregional only in 6 (6 %) patients, systemic only in 31 (30 %) patients, and combined in 14 (14 %) patients (electronic supplementary Table S2). Kaplan–Meier curves for disease-free survival are shown in Fig. 2.

## Discussion

This article presents our experience with RAMIE, using a new cohort, following our initial reports in 2006 and 2009 which showed this technique to be feasible and safe.^15^,^16^ In the current group of consecutive patients we focused on oncologic long-term follow-up. RAMIE was shown to be effective, with a high percentage of R0 radical resections (95 %) and adequate lymphadenectomy. RAMIE provided local control, with a low percentage of local recurrence. The high percentage of radical resections in our cohort with a majority of locally advanced T3 tumors (60 %) may be the result of the robotic surgical approach. Mainly, the 3D, magnified surgical view combined with the high degree of freedom of the articulating surgical instruments, facilitates precise dissection in a confined operating space.^18^

Nodal-positive disease (pN+) was observed in 56 % of all patients. A proper mediastinal lymphadenectomy was performed, including the right-sided paratracheal (lymph node station 2R), tracheobronchial (station 4), aortopulmonary window (station 5), carinal (station 7), and perioesophageal (station 8) lymph nodes, with a median of 26 dissected lymph nodes. This number is comparable to a series of open transthoracic esophagectomies from the literature.^6^ For conventional MIE, the median number of dissected lymph nodes was 21. Overall survival of patients who underwent RAMIE was comparable to the results following MIE.^26^,^27^

For recurrence, in this study the results following RAMIE with 65 % neoadjuvant treatment were comparable with the results reported for open esophagectomy, in which all patients received neoadjuvant chemoradiotherapy.^28^ The first site of symptomatic tumor recurrence was locoregional, or in the locoregional lymph nodes, in only 6 % of all cases. This is comparable with results after chemoradiotherapy, where locoregional recurrence was observed in 7 % of all cases.^28^ Distant metastases were observed in 30 % of all patients compared with 28 % for patients who underwent neoadjuvant chemoradiotherapy.^28^ The percentage of patients who had simultaneous locoregional recurrence and systemic metastases was 14 % in our cohort and 13 % after neoadjuvant chemoradiotherapy.^28^

Pneumonia was the most observed complication following RAMIE (34 % of patients). We compared our results with a recent randomized controlled trial where patients with resectable esophageal cancer were randomized between neoadjuvant chemoradiation and surgery alone. In this trial, only open esophagectomies were included, showing a pneumonia rate of 44 %.^20^ Another recent randomized controlled trial compared conventional MIE with open transthoracic esophagectomy.^14^ Results from this trial showed a reduced pulmonary complication rate in the MIE group compared with the open group.^14^ The percentage of in-hospital pulmonary infections after MIE in that trial was lower (12 %) than in our study;^14^ however, different definitions of postoperative pneumonia were used. Our definition of pneumonia was defined as the decision to treat suspected pneumonia (MCDC, grade II),^23^ while the definition of pneumonia used in the randomized controlled trial was more strict (infiltrate on pulmonary radiography combined with a positive sputum culture), leading to a lower percentage of pneumonia. Applying this definition to our cohort yields a pneumonia rate of 18 %, which is comparable to MIE.^14^ Reporting of postoperative pneumonia and postoperative outcomes after esophagectomy in general are heterogeneous and inconsistent. This makes comparison between different studies difficult and a consensus approach to reporting clinical outcomes should be considered.^29^,^30^

In addition to the aforementioned advantages of RAMIE, there were also disadvantages of RAMIE, such as the high costs of acquisition of the Da Vinci surgical system, disposable instruments, and a prolonged operative time compared with open esophagectomy.^18^ The introduction of RAMIE in a hospital needs careful proctoring by surgeons skilled and trained in RAMIE to reduce postoperative complications and to facilitate a steep learning curve.^15^ Centralization of robotic surgery in high-volume centers leads to a lower rate of postoperative complications and more efficient use of operating time.^31^

In this article we describe a decrease in thoracoscopic operative time between the first group of 43 patients and the second group of 42 patients (199 min vs. 166 min, respectively; *p* < 0.001), emphasizing the learning curve. The median duration of the full procedure is 381 min. We are currently performing the RAMIE procedure within 6 h. Furthermore, a significant decrease in the percentage of conversions was observed between the first group of 54 patients and the second group of 54 patients (13 [24 %] vs. 7 [13 %], respectively; *p* < 0.001). Currently our RAMIE conversion RATE is 4 %.

Our results from robot-assisted esophagectomy are in concordance with a recently published systematic review,^18^ which included nine articles (130 cases) describing robot-assisted esophagectomy. The level of evidence for RAMIE was suboptimal and was based on case series or expert opinions only (level 4 or 5).^18^ The aforementioned systematic review strongly emphasized the need for well-conducted randomized controlled trials and long-term survival studies within a framework of measured and comparable outcomes to prove the superiority of RAMIE over the worldwide current standard of open transthoracic esophagectomy.^18^ Therefore, we initiated the ROBOT trial (ClinicalTrial.gov identifier: NCT01544790) in January 2012 to compare RAMIE with open transthoracic esophagectomy.^32^

## Conclusions

In a cohort of Western European patients with advanced esophageal cancer, RAMIE with two-field lymphadenectomy was shown to be feasible and safe. Furthermore, RAMIE was shown to be oncologically effective, with a high percentage of R0 radical resections with adequate lymphadenectomy. RAMIE provided adequate local control, with a low percentage of local recurrence.

## Electronic Supplementary Material

Supplementary material 1 (DOCX 141 kb)
