# Supplementary material for: Oncologic Long-Term Results of Robot-Assisted Minimally Invasive Thoraco-Laparoscopic Esophagectomy with Two-Field Lymphadenectomy for Esophageal Cancer
Source: Ann Surg Oncol. 2015 May 29;22:1350–6. doi: 10.1245/s10434-015-4544-x (PMC4686562; doi:10.1245/s10434-015-4544-x)
Supplement: Supplementary file 1 — Supplementary material 1 (DOCX 141 kb) [file 10434_2015_4544_MOESM1_ESM.docx]

**Supplemental Figures and Tables**


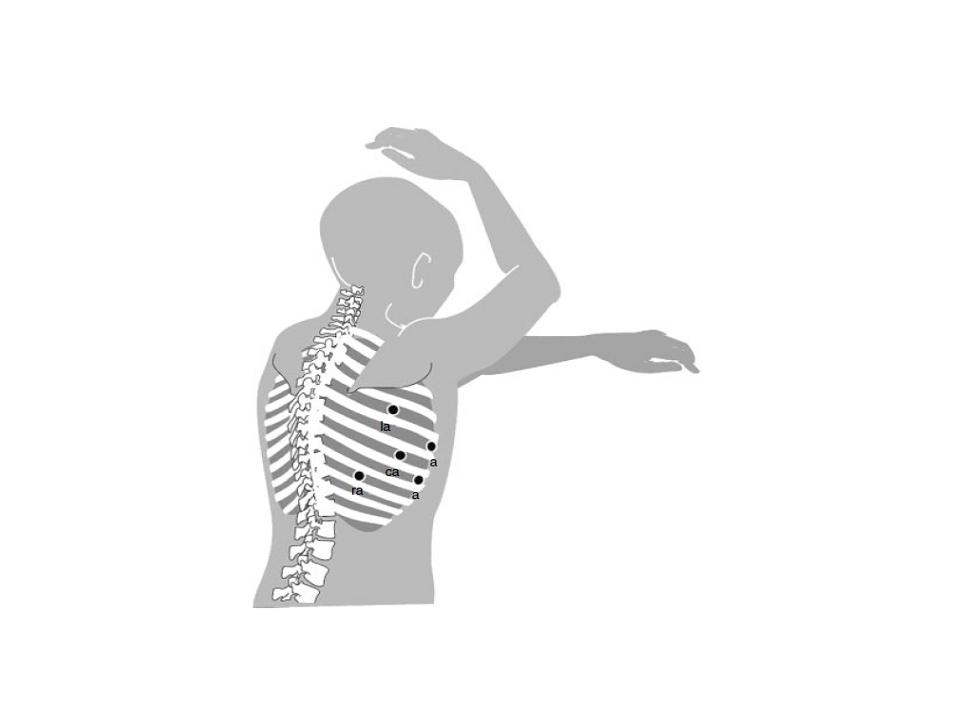

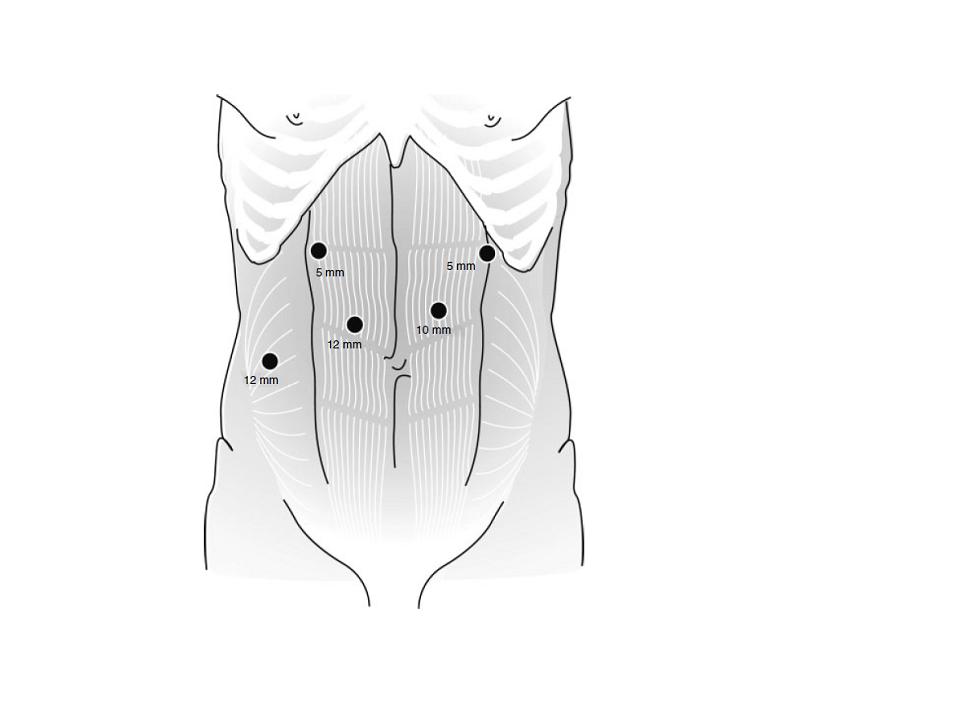


**S1a S1b**

**Figure S1. Trocar arrangement during the (a) robot-assisted thoracoscopic phase and (b) laparoscopic phase.^15^**

**Figure S1a.** Trocar arrangement during robot-assisted thoracoscopic phase. La: Left robotic arm (4^th^ intercostal space); a: assistant thoracoscopic working port (5^th^ and 7^th^ intercostal space); ca: robotic camera arm (6^th^ intercostal space); ra: right robotic arm (8^th^ or 9^th^ intercostal space).

**Figure S1b.** Trocar arrangement during the laparoscopic phase. The camera was inserted through the 10-mm para-umbilical trocar port and two 5-mm trocars were used as laparoscopic working ports. The liver retractor was inserted through the 12-mm right para-rectal trocar port. The harmonic ace was inserted through the 12-mm paraumbilical port. (with permission Boone et al).


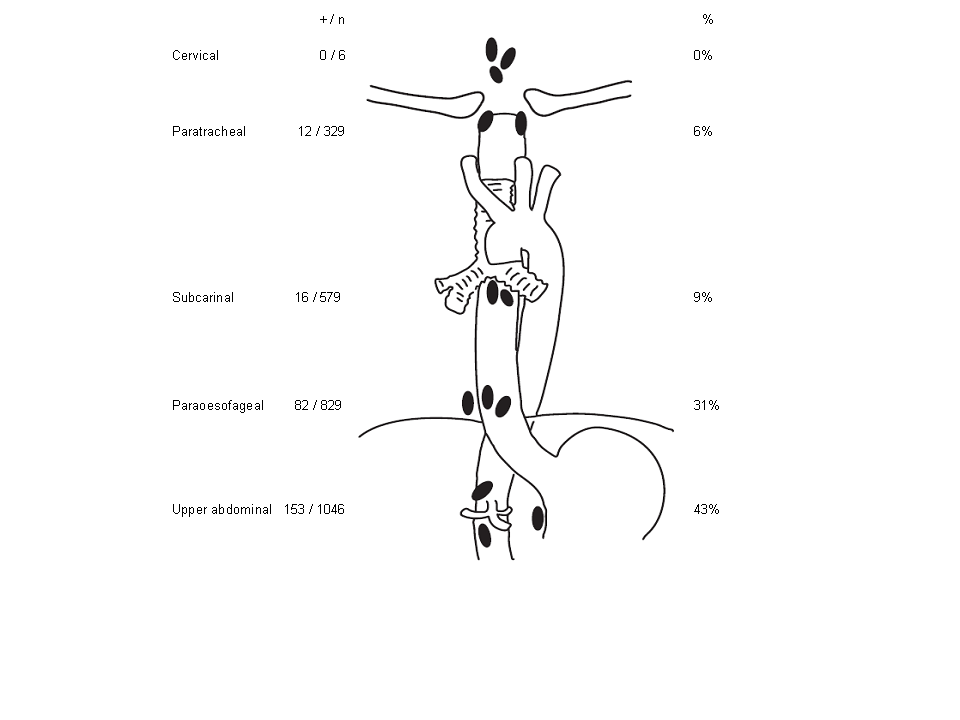


**Figure S2. Distribution of resected lymph nodes. Total number of lymph nodes (n), number of positive lymph nodes (+) and percentage of positive lymph nodes (%) at each location out of 85 patients.**

| **Table S1: Patient demographics and tumor characteristics** | | **(n=108)** |  |
| --- | --- | --- | --- |
|  | n (%) | Median | Range |
| **Age (y)** |  | 62 | 42 - 78 |
| **Gender** |  |  |  |
| M | 76 (70) |  |  |
| F | 32 (30) |  |  |
| **BMI (kg / m^2^)** |  | 26 | 16 – 36 |
| **Co-morbidity** |  |  |  |
| Vascular | 36 (33) |  |  |
| Cardiac | 25 (23) |  |  |
| Pulmonal | 15 (14) |  |  |
| Oncologic | 15 (14) |  |  |
| Previous thoracic / abdominal operation | 29 (27) |  |  |
| **ASA score** |  |  |  |
| 1 | 25 (23) |  |  |
| 2 | 74 (69) |  |  |
| 3 | 9 (8) |  |  |
| **Clinical T stage** |  |  |  |
| cT1 | 12 (11) |  |  |
| cT2 | 12 (11) |  |  |
| cT3 | 77 (71) |  |  |
| cT4 | 7 (7) |  |  |
| **Ultrasound N stage** |  |  |  |
| cN0 | 35 (32) |  |  |
| cN1-N3 | 83 (68) |  |  |
| **Tumor type** |  |  |  |
| Adenocarcinoma | 85 (79) |  |  |
| Squamous cell carcinoma | 23 (21) |  |  |
| **Neoadjuvant treatment** |  |  |  |
| No therapy | 39 (36) |  |  |
| Chemotherapy | 61 (57) |  |  |
| Chemoradiotherapy | 7 (7) |  |  |
| Radiotherapy | 1 (1) |  |  |

| **Table S2: Recurrence patterns after RAMIE (n=103)** | |
| --- | --- |
|  | n (%) |
| **No Recurrence** | 52 (50) |
| **Recurrence** | 51 (50) |
| **Locoregional*** | 6 (6) |
| **Distant^#^** | 31 (30) |
| **Both^$^** | 14 (14) |

* Recurrence of primary tumor or tumor in locoregional lymph nodes

# Hematogenous distant metastases or distant lymph node metastases

^$^ Synchronous locoregional recurrence and distant metastases
